# Evidence of multiple colonizations as a driver of black fly diversification in an oceanic island

**DOI:** 10.1371/journal.pone.0202015

**Published:** 2018-08-10

**Authors:** Yann Gomard, Josselin Cornuault, Séverine Licciardi, Erwan Lagadec, Boutaïna Belqat, Najla Dsouli, Patrick Mavingui, Pablo Tortosa

**Affiliations:** 1 Université de La Réunion, UMR PIMIT (Processus Infectieux en Milieu Insulaire Tropical), INSERM 1187, CNRS 9192, IRD 249, Plateforme Technologique CYROI, Sainte-Clotilde, La Réunion, France; 2 Department of Biodiversity and Conservation, Real Jardín Botánico, RJB-CSIC, Madrid, Spain; 3 CIRAD, UMR ASTRE, Sainte-Clotilde, La Réunion, France; 4 ASTRE, Univ Montpellier, CIRAD, INRA, Montpellier, France; 5 Groupement d’Intérêt Public Cyclotron Reunion Océan Indien (GIP CYROI), Sainte-Clotilde, La Réunion, France; 6 Department of Biology, Faculty of Sciences, University Abdelmalek Essaâdi, Tétouan, Morocco; 7 Centre de Recherche et de Veille sur les maladies émergentes dans l’Océan Indien (CRVOI), Plateforme Technologique CYROI, Sainte-Clotilde, La Réunion, France; 8 Université de Lyon, Lyon, France; Université Lyon 1, Villeurbanne, France; CNRS, UMR 5557, Ecologie Microbienne, Villeurbanne, France; INRA, UMR1418, Villeurbanne, France; Onderstepoort Veterinary Institute, SOUTH AFRICA

## Abstract

True oceanic islands typically host reduced species diversity together with high levels of endemism, which make these environmental set-ups ideal for the exploration of species diversification drivers. In the present study, we used black fly species (Diptera: Simuliidae) from Reunion Island as a model to highlight the main drivers of insect species diversification in this young and remote volcanic island located in the Southwestern Indian Ocean. Using local and regional (Comoros and Seychelles archipelagos) samples as well as specimens from continental Africa, we tested the likelihood of two distinct scenarios, *i*.*e*. multiple colonizations *vs*. *in-situ* diversification. For this, posterior odds were used to test whether species from Reunion did form a monophyletic group and we estimated divergence times between species. Three out of the four previously described Reunion black fly species could be sampled, namely *Simulium ruficorne*, *Simulium borbonense* and *Simulium triplex*. The phylogenies based on nuclear and mitochondrial markers showed that *S*. *ruficorne* and *S*. *borbonense* are the most closely related species. Interestingly, we report a probable mitochondrial introgression between these two species although they diverged almost six million years ago. Finally, we showed that the three Reunion species did not form a monophyletic group, and, combined with the molecular datation, the results indicated that Reunion black fly diversity resulted from multiple colonization events. Thus, multiple colonizations, rather than *in-situ* diversification, are likely responsible for an important part of black fly diversity found on this young Darwinian island.

## Introduction

Species assemblages within a community may gain new species through immigration or *in-situ* diversification [1,2]. The relative importance of each of these two processes in explaining local diversity is however subject to debate [3–7]. Islands and archipelagos represent highly favorable systems for understanding how local diversity arises and to describe evolutionary drivers at play [8–10]. Due to their geographic isolation, island systems are rather closed environments in which both species colonization and gene flow with other islands and/or continents are reduced, which is expected to favor local radiation [5,8,11,12]. Furthermore, despite their often-small size, islands generally harbor a diversity of habitats promoting sometimes exceptionally prolific diversification through adaptive radiation (e.g. spiders [13], anoles [14] and Darwin’s finches [15]). *In-situ* radiation may be further promoted in oceanic islands of volcanic origin, which generates topologically complex landscapes and thus varied habitats with limited inter-habitat gene flow [16,17].

**Fig 1:**
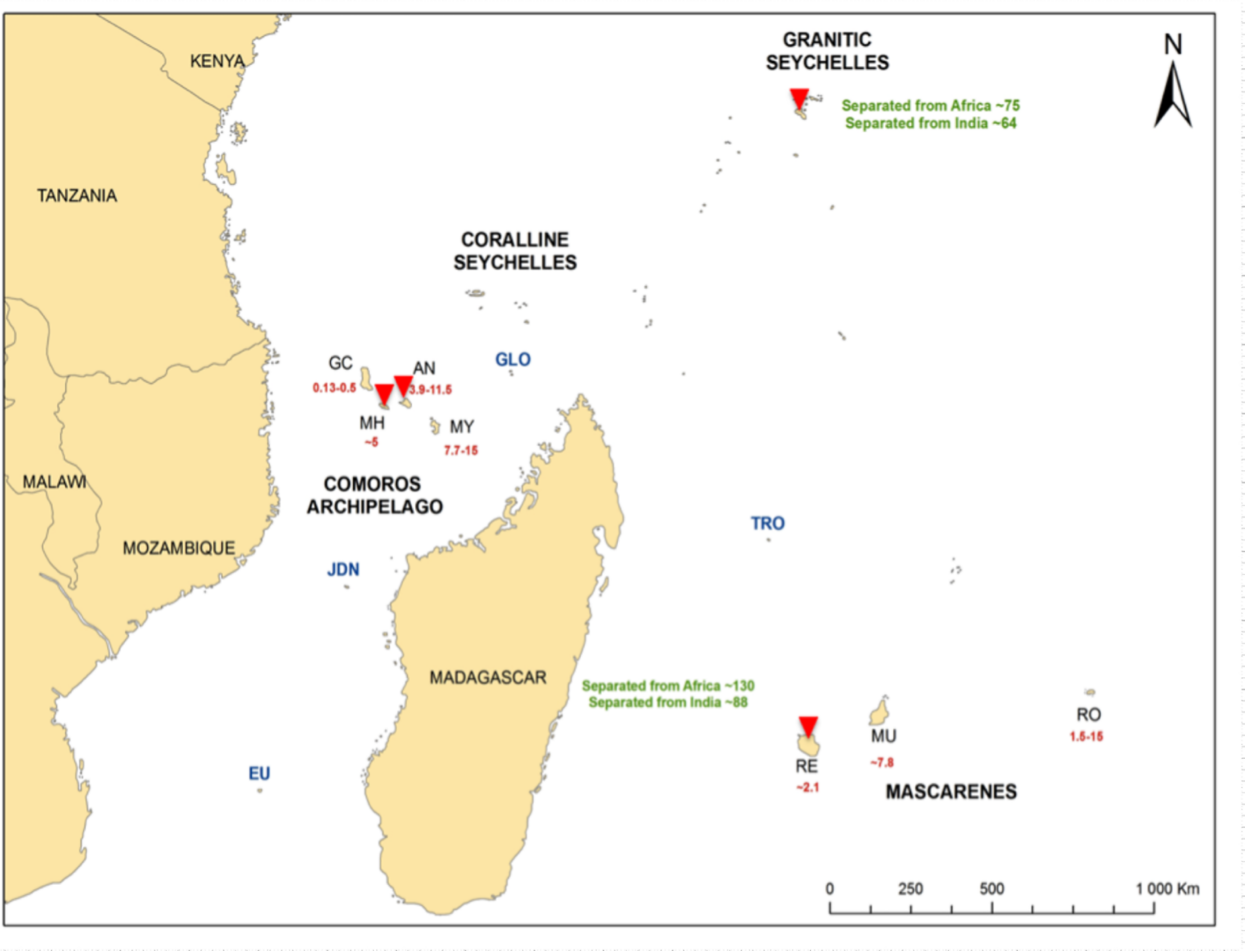
Geological context of the Southwestern Indian Ocean. Sample locations of black fly species are indicated by red triangles. The numbers correspond to island age in millions of years. GC: Grande Comore, MH: Mohéli, AN: Anjouan and MY: Mayotte, GLO: Glorieuses, JDN: Juan de Nova, EU: Europa, TRO: Tromelin, RE: Reunion Island, MU: Mauritius, RO: Rodrigues. Source map: ESRI World modified from Warren et al. [29].

Located in the Southwestern Indian Ocean (SWIO) region, Reunion Island is a young island, 2.1 million years (Myr), belonging to the Mascarene archipelago, which also includes Mauritius and Rodrigues islands (Fig 1). The SWIO basin also comprises Madagascar, Comoros and Seychelles archipelagos and forms one of the world’s 34 biodiversity hotspots [18]. These islands differ in their age, geological origin and distance to continent landmasses. As a result, biota hosted by these islands have different evolutionary histories and are appropriate for biogeographic and phylogeographic studies (see Agnarsson and Kuntner [19]). Located 800 km East of Madagascar, the Mascarene archipelago is the most remote archipelago of this insular ecosystem and is characterized by a high level of endemism [20]. In addition, Mascarene islands are young and considered as “Darwinian” islands (as defined by Gillespie and Roderick [5]) that have emerged *de novo* on volcanic hotspots. Hence, these islands have never been connected to continental masses and constitute suitable systems for investigating evolutionary processes involved in the assembly of species communities, as illustrated by several studies using plants [21,22], reptiles [23,24], nematodes [25], birds [26] and avian blood parasites as model organisms [27,28].

Black flies (Simuliidae) are insects of both medical and veterinary importance [30,31]. Some species transmit parasites such as the filarial *Onchocerca volvulus* (responsible for river blindness in human populations) or avian blood parasites of the genus *Leucocytozoon* [32–34]. The biting of adult females can also impair cattle and poultry productivity [35,36]. Furthermore, black flies play an important role in ecological processes, as their larvae constitute an important source of food for other organisms and participate to the treatment of organic materials in streams [31,37,38]. Interestingly, the Simuliidae family is highly diversified with more than 2,200 living species distributed on all continents except Antarctica [39]. Black flies are notably present on several remote islands and are hence interesting models for evolutionary studies on islands and archipelagos [40–46]. The SWIO islands house several black fly species, some of them with widespread geographic distributions whereas others are considered as endemics [39]. On Reunion Island, four species, belonging to the *ruficorne* species-group (subgenus *Nevermannia*), have been previously described based on morphological characters by Giudicelli [47]. *Simulium ruficorne* (Macquart, 1838) was originally described from Reunion Island while this species is currently known to have a large spatial distribution extending from the SWIO (Africa, Madagascar, Comoros and Mascarene archipelagos) to the Middle East and the Mediterranean sea [39,48]. Three additional species have been morphologically described using samplings carried out in 1983: *Simulium borbonense* (Giudicelli, 2008), *Simulium indoceanicum* (Giudicelli, 2008) and *Simulium triplex* (Giudicelli, 2008). These three species were considered as endemic to Reunion Island although *S*. *triplex* was later reported on Mauritius Island [39]. Based on morphological examinations, Giudicelli [47] suggested that the local black fly diversity results from successive colonization events from African continental *S*. *ruficorne* population. According to this hypothesis, each colonization has conducted to reproductive isolation before the arrival of another group of immigrants from African continental *S*. *ruficorne* population (multiple colonizations). This process would have taken place three times on Reunion Island.

In this study, we sequenced mitochondrial and nuclear genes from Reunion black fly species in order to investigate their phylogenetic relationships and evaluate divergence times between each of these species and African continental *S*. *ruficorne*. We then investigated the processes of diversification possibly involved by confronting alternative scenarios including (i) *in-situ* diversification within Reunion Island following a single colonization event of a *S*. *ruficorne* population and (ii) multiple independent colonizations.

## Materials and methods

### Ethics statement

No endangered or protected species was included in the present study. Most specimens were sampled on Reunion Island and did not require specific permits as the study did not include endangered species and did not involve sampling in protected areas. On Comoros archipelago, the samples were provided by an entomological survey carried out in the context of disease investigation for which the research protocol was approved by the Vice-Presidency of Agriculture, Fisheries and Environment of the Union of Comoros (see [49]). Samples from Morocco (Northern Africa) were provided by B. Belqat according to the permit delivered to the PhD student Y. EL Harym who was the collector.

### Sampling sites and specimens

The main sampling was conducted on Reunion Island from January to May 2011 (Fig 1 and Table 1). Larvae were collected in all ten perennial rivers of the island. For each river, two sampling stations were set up: a downstream station located below 40 meters above sea level, and, whenever possible, an upstream station located more than 500 meters above sea level. At each station, 60 larvae were sampled on submerged substrata as follows: 30 larvae were collected on submerged stones and 30 from submerged vegetation. Adults were captured using two methods. First, CDC miniature traps were mounted with UV leds and dry ice was placed in a container above the gear in order to feed the trap overnight with CO_2_. These traps were placed on the sampling sites (along or at the vicinity of a river) between 5.00 and 6.00 PM and picked up the next day between 7.00 and 8.00 AM. As this method lures the flies for a blood meal, mostly unfed females were captured. In addition, adults were captured using a butterfly net held out of a car driving at 30 Km.h^-1^ along perennial rivers. This method allowed trapping males and (fed/unfed) females. The sampling was opportunistically complemented with larvae specimens from other areas in the region (Comoros and Seychelles archipelagos) and from Africa (represented by larval specimens of *S*. *ruficorne* from Morocco) (Fig 1 and Table 1). All sampled specimens were immediately stored in 70° ethanol until laboratory analyses.

**Table 1:** Black fly specimens used in this study and GenBank accession numbers.

Designation	Subgenus	Specific epithet	Type	Location		Collection date	Coordinates		GenBank accession number	
							Latitude	Longitude	COI	18S/28S
GY018_borb	*Nevermannia*	*borbonense*	L	Reunion Island	Rivière Langevin, Saint-Joseph	09/01/11	21° 18' 41.472" S	55° 38' 29.256" E	JQ663445	JQ673499
GY022_borb	*Nevermannia*	*borbonense*	L	Reunion Island	Rivière Sainte-Etienne, Saint-Louis	10/01/11	21° 12' 25.524" S	55° 27' 1.908" E	JQ663446	NA
GY024_borb	*Nevermannia*	*borbonense*	L	Reunion Island	Rivière Langevin, Saint-Joseph	09/01/11	21° 18' 41.472" S	55° 38' 29.256" E	JQ663447	JQ673500
GY047_borb	*Nevermannia*	*borbonense*	L	Reunion Island	Rivière Langevin, Saint-Joseph	09/01/11	21° 18' 41.472" S	55° 38' 29.256" E	JQ663448	JQ673501
GY048_borb	*Nevermannia*	*borbonense*	L	Reunion Island	Rivière des Fleurs Jaunes, Salazie	20/01/11	21° 2' 1.212" S	55° 29' 37.644" E	JQ663449	JQ673502
GY049_borb	*Nevermannia*	*borbonense*	L	Reunion Island	Rivière des Fleurs Jaunes, Salazie	20/01/11	21° 2' 1.212" S	55° 29' 37.644" E	JQ663450	NA
GY050_borb	*Nevermannia*	*borbonense*	L	Reunion Island	Rivière des Fleurs Jaunes, Salazie	20/01/11	21° 1' 12.000" S	55° 32' 24.000" E	JQ663451	NA
GY053_borb	*Nevermannia*	*borbonense*	L	Reunion Island	Rivière des Fleurs Jaunes, Salazie	20/01/11	21° 1' 12.000" S	55° 32' 24.000" E	JQ663452	NA
GY062_borb	*Nevermannia*	*borbonense*	L	Reunion Island	Rivière Sainte-Etienne, Saint-Louis	10/01/11	21° 12' 25.524" S	55° 27' 1.908" E	JQ663453	NA
GY063_borb	*Nevermannia*	*borbonense*	L	Reunion Island	Rivière Sainte-Etienne, Saint-Louis	10/01/11	21° 12' 25.524" S	55° 27' 1.908" E	JQ663454	NA
GY077_borb	*Nevermannia*	*borbonense*	A	Reunion Island	Ravine du Chaudron, Sainte-Clotilde	22/02/11	20° 54' 36.000" S	55° 29' 24.000" E	JQ663455	NA
GY078_borb	*Nevermannia*	*borbonense*	A	Reunion Island	Ravine du Chaudron, Sainte-Clotilde	22/02/11	20° 54' 36.000" S	55° 29' 24.000" E	JQ663456	NA
GY079_borb	*Nevermannia*	*borbonense*	A	Reunion Island	Ravine du Chaudron, Sainte-Clotilde	22/02/11	20° 54' 36.000" S	55° 29' 24.000" E	JQ663457	NA
GY020_rufi	*Nevermannia*	*ruficorne*	L	Reunion Island	Rivière Sainte-Etienne, Saint-Louis	10/01/11	21° 17' 35.113" S	55° 24' 45.623" E	JQ663458	NA
GY025_rufi	*Nevermannia*	*ruficorne*	L	Reunion Island	Rivière Sainte-Etienne, Saint-Louis	10/01/11	21° 15' 25.524" S	55° 27' 32.544" E	JQ663459	NA
GY027_rufi	*Nevermannia*	*ruficorne*	L	Reunion Island	Rivière des Pluies, Sainte-Clotilde	07/01/11	20° 54' 15.480" S	55° 30' 19.800" E	JQ663460	NA
GY031_rufi	*Nevermannia*	*ruficorne*	L	Reunion Island	Rivière des Marsouins, Saint-Benoit	06/01/11	21° 2' 7.296" S	55° 42' 52.416" E	JQ663461	NA
GY035_rufi	*Nevermannia*	*ruficorne*	L	Reunion Island	Rivière du Mât, Saint-André	07/01/11	20° 58' 43.752" S	55° 41' 18.492" E	JQ663462	NA
GY040_rufi	*Nevermannia*	*ruficorne*	L	Reunion Island	Rivière Sainte-Etienne, Saint-Louis	10/01/11	21° 15' 25.524" S	55° 27' 32.544" E	JQ663463	JQ673503
GY041_rufi	*Nevermannia*	*ruficorne*	L	Reunion Island	Rivière des Galets, La Possession	10/01/11	20° 57' 43.704" S	55° 18' 35.352" E	JQ663464	JQ673504
GY042_rufi	*Nevermannia*	*ruficorne*	L	Reunion Island	Rivière des Galets, La Possession	10/01/11	20° 57' 43.704" S	55° 18' 35.352" E	JQ663465	JQ673505
GY051_rufi	*Nevermannia*	*ruficorne*	L	Reunion Island	Rivière des Fleurs Jaunes, Salazie	20/01/11	21° 1' 12.000" S	55° 32' 24.000" E	JQ663466	NA
GY056_rufi	*Nevermannia*	*ruficorne*	L	Reunion Island	Rivière des Fleurs Jaunes, Salazie	20/01/11	21° 1' 12.000" S	55° 32' 24.000" E	JQ663467	JQ673506
GY059_rufi	*Nevermannia*	*ruficorne*	L	Reunion Island	Rivière Sainte-Etienne, Saint-Louis	10/01/11	21° 17' 45.708" S	55° 24' 57.636" E	JQ663468	NA
GY061_rufi	*Nevermannia*	*ruficorne*	L	Reunion Island	Rivière Sainte-Etienne, Saint-Louis	10/01/11	21° 12' 25.524" S	55° 27' 1.908" E	JQ663469	JQ673507
GY067_rufi	*Nevermannia*	*ruficorne*	L	Reunion Island	Rivière des Fleurs Jaunes, Salazie	20/01/11	21° 2' 1.212" S	55° 29' 37.644" E	JQ663470	JQ673508
GY068_rufi	*Nevermannia*	*ruficorne*	L	Reunion Island	Rivière des Roches, Bras-Panon	06/01/11	21° 0' 20.736" S	55° 41' 38.252" E	JQ663471	NA
GY070_rufi	*Nevermannia*	*ruficorne*	L	Reunion Island	Rivière des Roches, Bras-Panon	06/01/11	21° 0' 20.736" S	55° 41' 38.252" E	JQ663472	JQ673509
GY075_rufi	*Nevermannia*	*ruficorne*	A	Reunion Island	Ravine du Chaudron, Sainte-Clotilde	22/02/11	20° 54' 36.000" S	55° 29' 24.000" E	JQ663473	NA
GY076_rufi	*Nevermannia*	*ruficorne*	A	Reunion Island	Ravine du Chaudron, Sainte-Clotilde	22/02/11	20° 54' 36.000" S	55° 29' 24.000" E	JQ663474	NA
GY080_rufi	*Nevermannia*	*ruficorne*	A	Reunion Island	Ravine du Chaudron, Sainte-Clotilde	22/02/11	20° 54' 36.000" S	55° 29' 24.000" E	JQ663475	NA
GY123_rufi	*Nevermannia*	*ruficorne*	A	Reunion Island	Rivière des Pluies, Sainte-Clotilde	09/03/11	20° 55' 12.000" S	55° 30' 36.000" E	JQ663476	NA
GY124_rufi	*Nevermannia*	*ruficorne*	A	Reunion Island	Rivière des Pluies, Sainte-Clotilde	09/03/11	20° 55' 12.000" S	55° 30' 36.000" E	JQ663477	NA
GY128_rufi	*Nevermannia*	*ruficorne*	A	Reunion Island	Sainte-Suzanne	09/03/11	20° 54' 59.8" S	55° 36' 30.5" E	JQ663478	NA
GY133_rufi	*Nevermannia*	*ruficorne*	A	Reunion Island	Rivière Langevin, Saint-Joseph	12/03/11	21° 22' 12.000" S	55° 38' 60.000" E	JQ663479	JQ673510
BB007_rufi	*Nevermannia*	*ruficorne*	L	Morocco (Northen Africa)	Séguia de Hay Aït Chaïb (Locality: Tazzarine, Province: Zagora)	17/06/15	30°46'48.03'' N	005°33'42.62'' W	KY421689	KY421701
BB008_rufi	*Nevermannia*	*ruficorne*	L	Morocco (Northen Africa)	Séguia de Hay Aït Chaïb (Locality: Tazzarine, Province: Zagora)	17/06/15	30°46'48.03'' N	005°33'42.62'' W	KY421690	KY421702
BB009_rufi	*Nevermannia*	*ruficorne*	L	Morocco (Northen Africa)	Séguia de Hay Aït Chaïb (Locality: Tazzarine, Province: Zagora)	17/06/15	30°46'48.03'' N	005°33'42.62'' W	KY421691	KY421703
BB010_rufi	*Nevermannia*	*ruficorne*	L	Morocco (Northen Africa)	Séguia de Hay Aït Chaïb (Locality: Tazzarine, Province: Zagora)	17/06/15	30°46'48.03'' N	005°33'42.62'' W	KY421692	KY421704
BB011_rufi	*Nevermannia*	*ruficorne*	L	Morocco (Northen Africa)	Séguia de Hay Aït Chaïb (Locality: Tazzarine, Province: Zagora)	17/06/15	30°46'48.03'' N	005°33'42.62'' W	KY421693	KY421705
BB012_rufi	*Nevermannia*	*ruficorne*	L	Morocco (Northen Africa)	Séguia de Hay Aït Chaïb (Locality: Tazzarine, Province: Zagora)	17/06/15	30°46'48.03'' N	005°33'42.62'' W	KY421694	KY421706
BB013_rufi	*Nevermannia*	*ruficorne*	L	Morocco (Northen Africa)	Séguia de Hay Aït Chaïb (Locality: Tazzarine, Province: Zagora)	17/06/15	30°46'48.03'' N	005°33'42.62'' W	KY421695	KY421707
BB014_rufi	*Nevermannia*	*ruficorne*	L	Morocco (Northen Africa)	Séguia de Hay Aït Chaïb (Locality: Tazzarine, Province: Zagora)	17/06/15	30°46'48.03'' N	005°33'42.62'' W	KY421696	KY421708
BB015_rufi	*Nevermannia*	*ruficorne*	L	Morocco (Northen Africa)	Séguia de Hay Aït Chaïb (Locality: Tazzarine, Province: Zagora)	17/06/15	30°46'48.03'' N	005°33'42.62'' W	KY421697	KY421709
BB016_rufi	*Nevermannia*	*ruficorne*	L	Morocco (Northen Africa)	Séguia de Hay Aït Chaïb (Locality: Tazzarine, Province: Zagora)	17/06/15	30°46'48.03'' N	005°33'42.62'' W	KY421698	KY421710
BB017_rufi	*Nevermannia*	*ruficorne*	L	Morocco (Northen Africa)	Séguia de Hay Aït Chaïb (Locality: Tazzarine, Province: Zagora)	17/06/15	30°46'48.03'' N	005°33'42.62'' W	KY421699	KY421711
BB018_rufi	*Nevermannia*	*ruficorne*	L	Morocco (Northen Africa)	Séguia de Hay Aït Chaïb (Locality: Tazzarine, Province: Zagora)	17/06/15	30°46'48.03'' N	005°33'42.62'' W	KY421700	KY421712
GY019_trip	*Nevermannia*	*triplex*	L	Reunion Island	Rivière Sainte-Etienne, Saint-Louis	10/01/11	21° 17' 35.113" S	55° 24' 45.623" E	JQ663480	NA
GY029_trip	*Nevermannia*	*triplex*	L	Reunion Island	Rivière des Remparts, Saint-Joseph	08/01/11	21° 22' 59.196" S	55° 37' 6.816" E	JQ663481	NA
GY030_trip	*Nevermannia*	*triplex*	L	Reunion Island	Rivière des Remparts, Saint-Joseph	08/01/11	21° 22' 59.196" S	55° 37' 6.816" E	JQ663482	NA
GY033_trip	*Nevermannia*	*triplex*	L	Reunion Island	Rivière des Roches, Bras-Panon	06/01/11	21° 0' 20.736" S	55° 41' 38.252" E	JQ663483	NA
GY034_trip	*Nevermannia*	*triplex*	L	Reunion Island	Rivière des Roches, Bras-Panon	06/01/11	21° 1' 21.180" S	55° 40' 9.768" E	JQ663484	NA
GY036_trip	*Nevermannia*	*triplex*	L	Reunion Island	Rivière du Mât, Saint-André	07/01/11	20° 58' 43.752" S	55° 41' 18.492" E	JQ663485	NA
GY038_trip	*Nevermannia*	*triplex*	L	Reunion Island	Rivière Langevin, Saint-Joseph	08/01/11	21° 23' 7.440" S	55° 38' 39.336" E	JQ663486	NA
GY039_trip	*Nevermannia*	*triplex*	L	Reunion Island	Rivière Langevin, Saint-Joseph	08/01/11	21° 23' 7.440" S	55° 38' 39.336" E	JQ663487	NA
GY052_trip	*Nevermannia*	*triplex*	L	Reunion Island	Rivière des Fleurs Jaunes, Salazie	20/01/11	21° 1' 12.000" S	55° 32' 24.000" E	JQ663488	JQ673511
GY057_trip	*Nevermannia*	*triplex*	L	Reunion Island	Rivière des Marsouins, Saint-Benoit	06/01/11	21° 2' 7.296" S	55° 42' 52.416" E	JQ663489	NA
GY058_trip	*Nevermannia*	*triplex*	L	Reunion Island	Rivière des Marsouins, Saint-Benoit	06/01/11	21° 2' 7.296" S	55° 42' 52.416" E	JQ663490	NA
GY060_trip	*Nevermannia*	*triplex*	L	Reunion Island	Rivière Sainte-Etienne, Saint-Louis	10/01/11	21° 17' 45.708" S	55° 24' 57.636" E	JQ663491	NA
GY064_trip	*Nevermannia*	*triplex*	L	Reunion Island	Rivière du Mât, Saint-André	07/01/11	20° 58' 43.752" S	55° 41' 18.492" E	JQ663492	NA
GY065_trip	*Nevermannia*	*triplex*	L	Reunion Island	Rivière des Fleurs Jaunes, Salazie	20/01/11	21° 2' 1.212" S	55° 29' 37.644" E	JQ663493	NA
GY069_trip	*Nevermannia*	*triplex*	L	Reunion Island	Rivière des Roches, Bras-Panon	06/01/11	21° 0' 20.736" S	55° 41' 38.252" E	JQ663494	NA
GY072_trip	*Nevermannia*	*triplex*	L	Reunion Island	Rivière des Roches, Bras-Panon	06/01/11	21° 0' 20.736" S	55° 41' 38.252" E	JQ663495	NA
GY073_trip	*Nevermannia*	*triplex*	L	Reunion Island	Rivière des Roches, Bras-Panon	06/01/11	21° 1' 21.180" S	55° 40' 9.768" E	JQ663496	NA
GY119_trip	*Nevermannia*	*triplex*	A	Reunion Island	Rivière des Pluies, Sainte-Clotilde	08/03/11	20° 55' 12.000" S	55° 30' 36.000" E	JQ663497	JQ673512
GY120_trip	*Nevermannia*	*triplex*	A	Reunion Island	Rivière des Pluies, Sainte-Clotilde	09/03/11	20° 55' 12.000" S	55° 30' 36.000" E	NA	JQ673513
GY121_trip	*Nevermannia*	*triplex*	A	Reunion Island	Rivière des Pluies, Sainte-Clotilde	09/03/11	20° 55' 12.000" S	55° 30' 36.000" E	JQ663498	NA
GY127_trip	*Nevermannia*	*triplex*	A	Reunion Island	Sainte-Suzanne	09/03/11	20° 54' 59.8" S	55° 36' 30.5" E	JQ663499	NA
SL001_como	*Nevermannia*	*triplex*	L	Comoros archipelago	Anjouan	24/02/11	12° 07' 38.5" S	44° 25' 51.5" E	JQ663500	NA
SL002_como	*Nevermannia*	*triplex*	L	Comoros archipelago	Anjouan	24/02/11	12° 07' 38.5" S	44° 25' 51.5" E	JQ663501	NA
SL003_como	*Nevermannia*	*triplex*	L	Comoros archipelago	Ouallah 1, Mohéli	24/02/11	12° 19' 39.432" S	43° 40' 7.428" E	JQ663502	NA
SL004_como	*Nevermannia*	*triplex*	L	Comoros archipelago	Ouallah 1, Mohéli	24/02/11	12° 19' 39.432" S	43° 40' 7.428" E	JQ663503	JQ673514
GY239_seyc	*Simulium* sp. 1		L	Seychelles archipelago	Port Glaud Waterfall, Mahé	05/04/11	4° 39' 30.44" S	55° 24' 45.76" E	JQ663504	JQ673515
GY240_seyc	*Simulium* sp. 1		L	Seychelles archipelago	Port Glaud Waterfall, Mahé	05/04/11	4° 39' 30.44" S	55° 24' 45.76" E	JQ663505	NA
GY241_seyc	*Simulium* sp. 1		L	Seychelles archipelago	Port Glaud Waterfall, Mahé	05/04/11	4° 39' 30.44" S	55° 24' 45.76" E	JQ663506	NA
GY242_seyc	*Simulium* sp. 1		L	Seychelles archipelago	Port Glaud Waterfall, Mahé	05/04/11	4° 39' 30.44" S	55° 24' 45.76" E	JQ663507	NA
*S*. aureohirtum*	*Nevermannia*	*aureohirtum*		Thailand					JF916850	NA
*S*. aureohirtum*	*Nevermannia*	*aureohirtum*		Thailand					KF289401	NA
*S*. aureohirtum*	*Nevermannia*	*aureohirtum*		China					KP793690	NA
*S*. aureohirtum*	*Nevermannia*	*aureohirtum*		China					NA	FJ538878
*S*. aureohirtum*	*Nevermannia*	*aureohirtum*		China					NA	FJ538879
*S*. aureohirtum*	*Nevermannia*	*aureohirtum*		China					NA	FJ538881
*S*. aureohirtum*	*Nevermannia*	*aureohirtum*		China					NA	FJ538882
*S*. aureohirtum*	*Nevermannia*	*aureohirtum*		China					NA	FJ538883
*S*. aureohirtum*	*Nevermannia*	*aureohirtum*		China					NA	FJ538884
*S*. aureohirtum*	*Nevermannia*	*aureohirtum*		China					NA	FJ538886
*S*. aureohirtum*	*Nevermannia*	*aureohirtum*		China					NA	FJ538887
*S*. aureohirtum*	*Nevermannia*	*aureohirtum*		China					NA	FJ538888
*S*. aureohirtum*	*Nevermannia*	*aureohirtum*		China					NA	FJ538889
*S*. feuerborni*	*Nevermannia*	*feuerborni*		Thailand					JX484813	NA
*S*. fruticosum*	*Nevermannia*	*feuerborni*		Thailand					KF289399	NA
*S*. merga*	*Montisimulium*	*merga*		Thailand					KF289396	NA
*S*. pahagense*	*Daviesellum*	*pahangense*		Thailand					KF289460	NA
*S*. quinquestriatum*	*Simulium*	*quinquestriatum*		China					JQ412151	NA
*S*. bidentatum*	*Simulium*	*bidentatum*		China					DQ534947	NA

L: Larvae; A: Adult; NA: Not Available. The asterisks indicate species which were not sampled in the present study. For these species, only information relative to countries and GenBank accession numbers are provided.

### Morphological and molecular identification

All Reunion black fly larvae could be identified morphologically using previously published keys [47]. For larvae, three specific morphological characteristics were used: ventral and dorsal ornamentations on the head capsule together with hypostomium shape. To improve identification of larvae and adults, a potential size polymorphism in the Internal Transcribed Spacer 1 (ITS1) was investigated as this nuclear marker has been shown to be variable in size between closely related black fly species [50,51]. Using DNA from morphologically identified larvae as template; a Polymerase Chain Reaction (PCR) amplification of the ITS1 locus was carried out using previously published primers, ITS1-5’/ITS1-3’ [52] (see *DNA extraction*, *amplification and sequencing* section). Differences in amplicon size were visualized by electrophoresis on 2% agarose gels stained with 1X GelRed^TM^ (Biotium Inc.) and further confirmed by Sanger sequencing.

### DNA extraction, amplification and sequencing

A sample of 14 adults and 62 larvae (comprising at least one larva per species and sampling station in Reunion) was used for molecular analyses. DNA extraction was realized with the Qiagen EZ1 DNA Tissue kit (Qiagen Corporation, Valencia, CA) according to the manufacturer's protocol. The mitochondrial cytochrome *c* oxidase subunit I encoding gene (COI) and a nuclear fragment encompassing 18S rDNA (3’ end), Internal Transcribed Spacer 1 (ITS1), 5.8S rDNA, Internal Transcribed Spacer 2 (ITS2) and 28S rDNA (5’ end) *locus* (thereafter referred as 18S/28S) was amplified and sequenced for phylogenetic reconstructions. COI was amplified with LCOI490/HCO2198 primers [53] and 18S/28S with specific primers described in Brockhouse et al. [54]. Amplification conditions for COI consisted of an initial denaturation step at 94°C for 3 min followed by 30 cycles at 94°C for 30 s, 50°C for 30 s and 72°C for 1 min, and by a final elongation step of 7 min at 72°C. Ribosomal DNA, both 18S/28S and ITS1, were amplified using the following conditions: 3 min at 94°C, followed by 40 cycles of 30 sec at 95°C, 50 sec at 45°C and 50 sec at 72°C. The amplification ended with a final step of 7 min at 72°C. Bi-directional sequencing was carried out using the amplifying primers. Whenever nuclear sequences revealed heterozygote specimens, amplicons were gel-purified and cloned into a pGEM-T Easy Vector (Promega Corporation, Fitchburg, WI). Four randomly selected transformants were then sequenced in order to get the sequences of both alleles. All COI and 18S/28S sequences were deposited in GenBank under JQ663445-JQ663507, JQ673499-JQ673515 and KY421689-KY421712 accession numbers (Table 1).

### Phylogenetic analyses

Sequences were aligned using MAFFT implemented in Geneious pro software v.5.4 [55]. Additional black fly species sequences from GenBank were integrated in phylogenies as potential close relatives, including in particular available sequences of *Simulium aureohirtum* (Brunetti, 1911) (*ruficorne* species-group) from Asia (Table 1).

Dated phylogenetic trees were estimated with BEAST v2.3.2 [56] for nuclear and mitochondrial data independently but using the same model, described here. The substitution model used was the GTR + G + I, in which all other substitution models are nested. Relative exchangeability rates were assigned a Gamma (shape = 1,scale = 1) prior. Equilibrium base frequencies were estimated. Among-site heterogeneity in substitution rates (+ G) was accommodated with the inclusion of site-specific substitution rate multipliers. These multipliers were assigned a Gamma (shape = alpha, rate = alpha) prior, with alpha fixed to 11.1. This parametrization of the gamma prior implies a prior coefficient of variation of substitution rates among sites of ~ 0.3. Rate multipliers were integrated out of the model using a discrete approximation of the gamma prior [57] with six categories. The proportion of invariant sites (+ I) was assigned a Uniform (0,1) prior. An uncorrelated log-normal relaxed molecular clock was used, with a fixed mean (ucldMean parameter) and a free standard deviation (ucldStdev) parameter with a Gamma (shape = 0.5396, scale = 0.3819) prior. This parametrization of the ucldStdev prior implies that 97.5% of the prior density is in favour of a coefficient of variation of rates among branches less than 1. For the mitochondrial analysis, ucldMean was fixed to the standard mutation rate of arthropods, *i*.*e*. 0.0115 substitutions site^-1^ Myr^-1^ [58]. For the nuclear analysis, since we had no information about the substitution rate, we fixed ucldMean to 0.004, a value yielding a phylogenetic tree of roughly the same height as for the mitochondrial analysis. This makes the time unit of the nuclear tree roughly similar to that of the mitochondrial DNA tree, so that the same priors for time-related parameters can be used in both analyses. However, the time-scale of the nuclear tree should not be interpreted. A birth-death tree prior was used, with a Lognormal (1,1.25) for the speciation rate (birthRate2) and a Beta (1,2) prior for the relative extinction rate (*i*.*e*. the extinction-to-speciation rates ratio, relativeDeathRate2). For both the mitochondrial and nuclear analyses, two independent MCMC chains were run for a total of 30 million generations each, of which 10% were discarded as burn-in. Samples were kept every 1,000^th^ generation. Convergence was verified by effective sample size (ESS) values exceeding 200 for all parameters, using Tracer v1.6 for continuous parameters. For the tree topology parameter, we calculated the pseudo- and approximate ESSs, as implemented in the functions *topological*.*pseudo*.*ess* and *topological*.*approx*.*ess* of the R package *rwty* [59], based on the path distance between trees. The posterior sample of trees was summarized into a unique maximum-clade-credibility consensus tree with posterior median node ages using TreeAnnotator v2.3.2.

Sequences were available for both nuclear and mitochondrial markers only for a small number of individuals. Consequently, we chose to keep both nuclear and mitochondrial analyses separate.

### Monophyly tests

In order to assess the likelihood of a scenario with a single colonization of Reunion by black flies, with a subsequent *in-situ* diversification, we assessed the likelihood of all Reunion species forming a monophyletic clade. As the Comorian specimens were identified as *S*. *triplex* (see the *Results* section), these specimens were synonymized with Reunion *S*. *triplex* in this monophyly analysis, effectively testing a hypothesis of *in-situ* diversification in the SWIO region (Hypothesis *H*_*1*_) rather than on Reunion Island alone.

We further evaluated the monophyly of Reunion *S*. *ruficorne* specimens (Hypothesis *H*_*2*_), as the mitochondrial phylogeny suggested the paraphyly of *S*. *ruficorne* (see the *Results* section).

Based on Bergsten et al. [60], we did not use Bayes Factors to evaluate clade monophyly, given the difficulty to define appropriate prior probabilities of hypotheses. Instead, each of the two hypotheses (*H*_*1*_ and *H*_*2*_) was assessed using its posterior odd, calculated as: P(*H*|D) / (1-P(*H*|D)), where P(*H*|D) is the posterior probability of hypothesis *H* [60]. The posterior odd of a monophyly hypothesis indicates how many times more likely monophyly is relative to non-monophyly. P(*H*|D) was estimated as the frequency of trees in the posterior distribution that included the clade considered, when this frequency was more than 0. When the clade of interest is not comprised in any tree of the posterior sample (zero frequency), we estimated that P(*H*|D) was less than 1/ESS_topology_.

## Results

### Molecular and morphological identification

Sampling on Reunion Island allowed the collection of three out of the four previously described species, namely *Simulium ruficorne*, *Simulium borbonense* and *Simulium triplex*. No *Simulium indoceanicum* specimen was captured. For each sampled species, the amplification of the ITS1 *locus* produced amplicons with different sizes: 82, 110 and 116 bp for *S*. *triplex*, *S*. *ruficorne* and *S*. *borbonense*, respectively (Table 2). This size polymorphism can be visualized by electrophoresis on a 2% agarose gel and is thus usable for rapid molecular identification of Reunion black flies (S1 Fig). The amplification of the ITS1 *locus* produced amplicons of the same size for both Reunion and continental African *S*. *ruficorne* specimens (Table 2). Similarly, the amplification of the ITS1 *locus* from all Comorian specimens and Reunion *S*. *triplex* specimens produced amplicons of the same size (Table 2). Moreover, larvae sampled in Comoros could not be differentiated from *S*. *triplex* using morphological characters. These results together with the produced phylogeny (see below) identify black flies collected on Comoros as *S*. *triplex*. Thus the collected Comorian specimens will be referred to as *S*. *triplex*. Larvae from the Seychelles archipelago showed specific morphological characters and the ITS1 nucleotides sequence was clearly different from that obtained with specimens from Reunion, Africa or the Comoros, and therefore represent a species otherwise absent from our sample.

**Table 2:** Details on nuclear (ITS1 and 18S/28S) and mitochondrial (COI) sequences obtained from the black fly specimens of this study.

Location	Species	ITS1 *locus* size	Number of haplotypes detected for COI *locus* (658 bp)	Number of haplotypes detected for 18S/28S *locus* (576–650 bp)
Reunion	*S*. *triplex*	82 bp	1 (*n = 20*)	1 (*n = 3*)
	*S*. *ruficorne*	110 bp	10 (*n = 16*)	4 (*n = 8*)
	*S*. *borbonense*	116 bp	12 (*n = 19*)	2 (*n = 4*)
Africa	*S*. *ruficorne*	110 bp	6 (*n = 12*)	5 (*n = 12*)
Comoros	*S*. *triplex*	82 bp	1 (*n = 4*)	1 (*n = 1*)
Seychelles	*Simulium* sp. 1	111 bp	1 (*n = 4*)	1 (*n = 1*)

For 18S/28S and COI *locus* the numbers in brackets correspond to the number of produced sequences according to each species.

Based on the detected length polymorphism of ITS1, we also proposed additional discriminating morphological characters allowing identification of adults from Reunion. Both females and males of *S*. *triplex* have entirely black antennae except for a white first segment. In contrast, *S*. *borbonense* and *S*. *ruficorne* females have white antennae with a black first segment. These two latter species can be distinguished according to their legs: *S*. *borbonense* has a large black patch on each femur while *S*. *ruficorne* exhibits tiny spots. *Simulium borbonense and S*. *ruficorne* males could not be morphologically distinguished.

### Phylogenetic analysis

The amplification of the mitochondrial locus produced 75 sequences of 658 nucleotides without deletions or insertions. No polymorphism was found in mitochondrial sequences within Reunion (*n = 20*) or within Comorian (*n = 4*) *S*. *triplex*, nor within Seychelles specimens (*n = 4*) (Table 2). In contrast, mitochondrial sequences obtained from *S*. *borbonense* (*n = 19*) or from Reunion (*n = 16*) and African *S*. *ruficorne* (*n = 12*) showed nucleotide polymorphism comprising respectively 12, ten and six distinct haplotypes. Reunion and African *S*. *ruficorne* did not share any mitochondrial haplotype. Nuclear amplification (18S/28S) produced 29 sequences ranging from 576 to 650 bp for *S*. *borbonense* (*n = 4*), Reunion (*n = 3*) and Comorian (*n = 1*) *S*. *triplex*, Reunion (*n = 8*) and African (*n = 12*) *S*. *ruficorne* and only one sequence was obtained from a Seychelles specimen (Table 2). Interestingly, although *S*. *ruficorne* specimens from Reunion and continental Africa did not share any mitochondrial haplotype, they did share one nuclear sequence.

**Fig 2:**
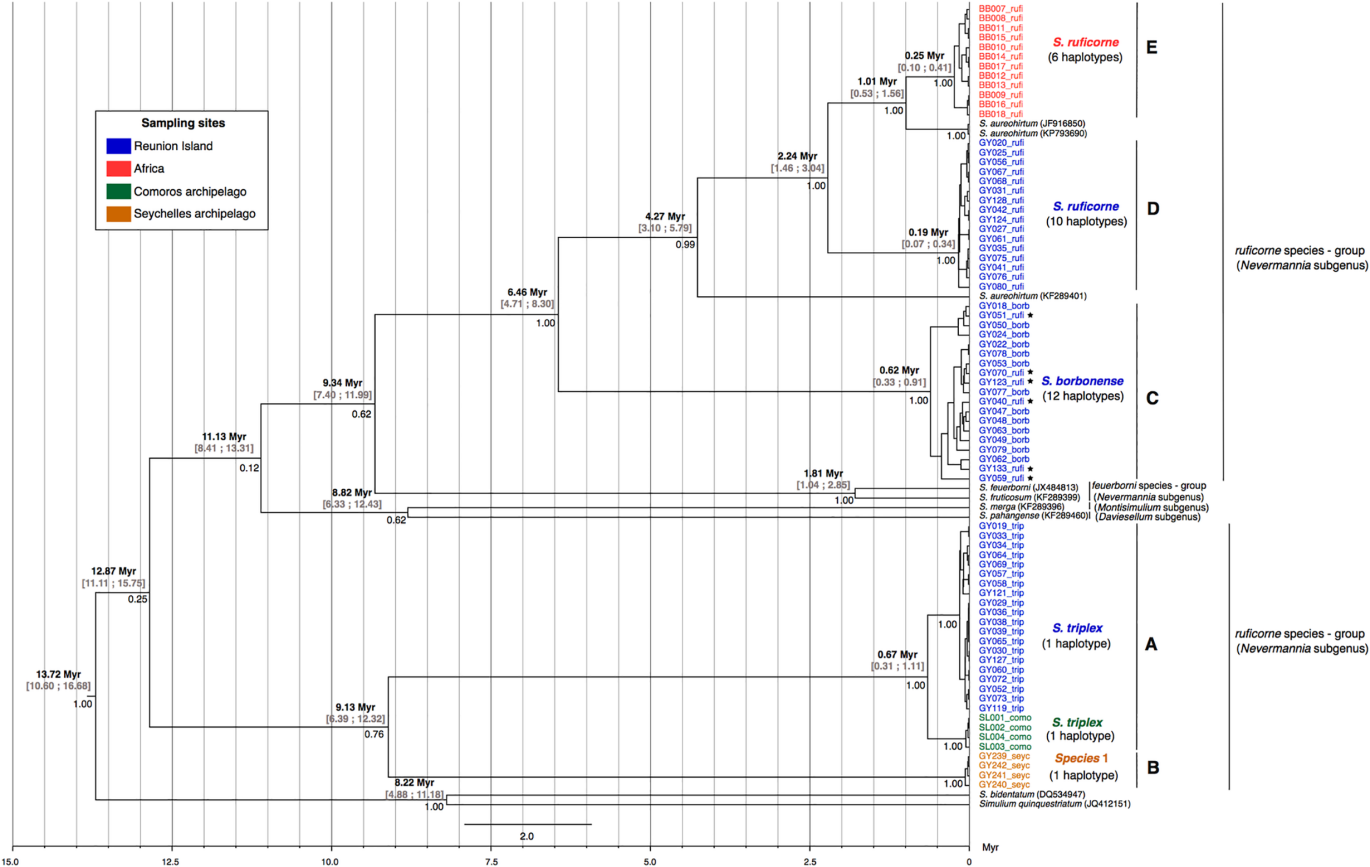
Phylogenetic tree based on mitochondrial sequences (COI, 658 bp) of black fly species from Reunion Island, Northen of Africa (Morocco), Comoros (Anjouan and Mohéli islands) and Seychelles archipelago (Mahé island). This analysis was carried out with BEAST v.2.3.2. Each sequence corresponds to one individual and the haplotype number is indicated between brackets. The estimated divergence times and posterior probabilities are indicated above and below the nodes, respectively. The time unit is per million year (Myr). Within the *Simulium borbonense* clade, the stars indicate specimens identified as Reunion *Simulium ruficorne* by morphology and the ITS1 diagnostic.

**Fig 3:**
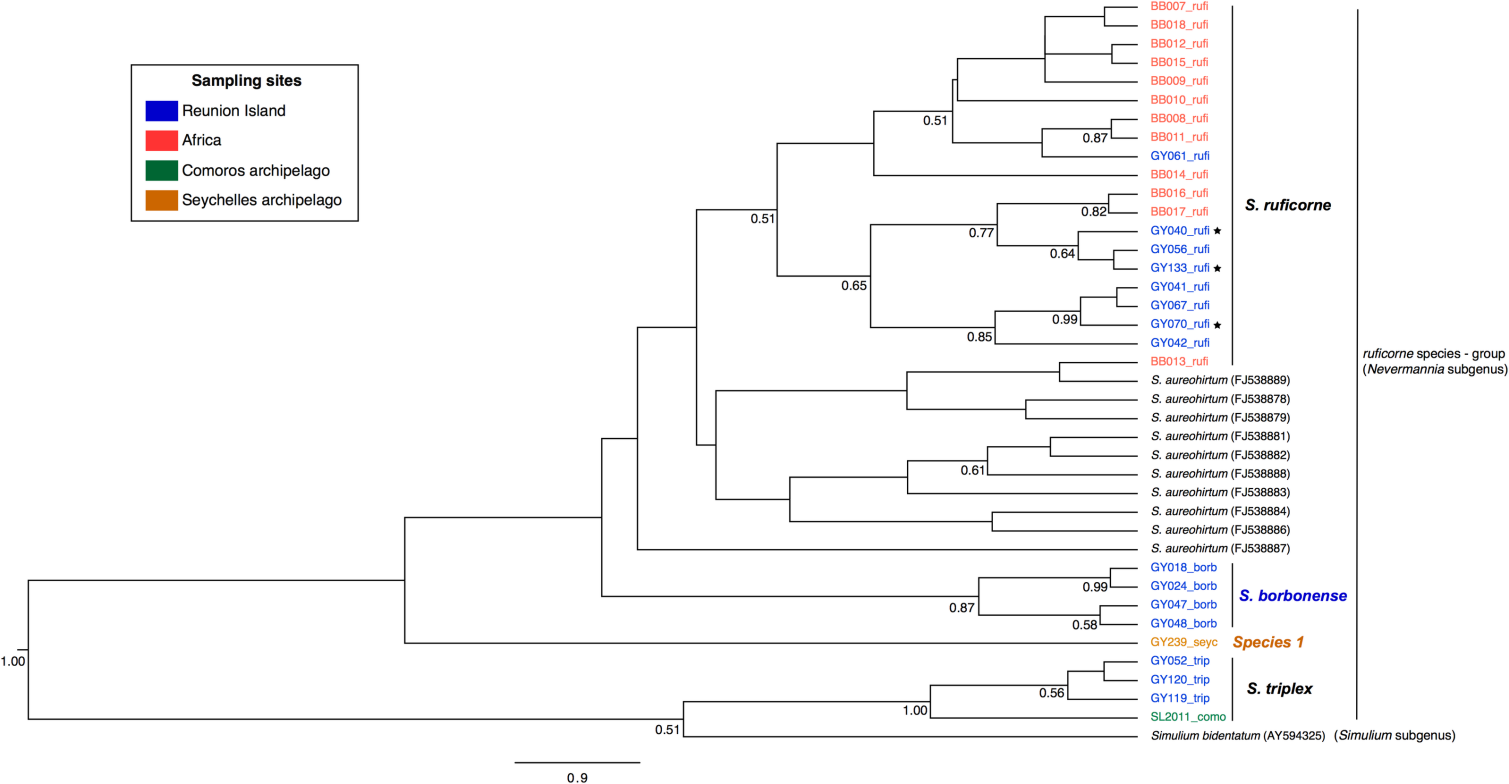
Phylogenetic tree based on nuclear sequences (18S/28S, 672 bp) of black fly species from Reunion Island, Northen of Africa (Morocco), Comoros (Anjouan and Mohéli islands) and Seychelles archipelago (Mahé island). This analysis was conducted with BEAST v.2.3.2. Each sequence corresponds to one specimen. The numbers at the nodes are posterior probabilities and only posterior probabilities superior to 0.50 are indicated. The time scale is unknown for nuclear markers. Within the clade of Reunion *Simulium ruficorne*, the stars indicate specimens with *S*. *borbonense* mitochondrial haplotypes (see Fig 2).

The mitochondrial phylogeny (Fig 2) confirmed the identification of Comorian specimens as *S*. *triplex*, which clustered with Reunion *S*. *triplex* (Clade A). In contrast, the specimens from Seychelles formed a clade of their own (Clade B), related to but quite divergent from *S*. *triplex*. All Reunion and African *S*. *ruficorne* grouped together. The *S*. *aureohirtum* specimens fell within the *ruficorne* species-group. Interestingly, a number of sequences obtained from Reunion *S*. *ruficorne* specimens (designated by stars in Fig 2) clustered with *S*. *borbonense* haplotypes in Clade C. The latter specimens were however confirmed as *S*. *ruficorne* based on morphology, ITS1 length polymorphism typing and the nuclear phylogeny (see Fig 3 and S1 Fig). The remaining sequences from Reunion *S*. *ruficorne* otherwise formed a monophyletic clade (Clade D), as did sequences from African *S*. *ruficorne* (Clade E).

The nuclear phylogeny (Fig 3) was less resolved than the mitochondrial one but confirmed the identification of Comorian specimens as *S*. *triplex*. The topology further showed that the *S*. *ruficorne* specimens harbouring *S*. *borbonense* haplotypes (designated by stars in Fig 3) possessed *S*. *ruficorne* nuclear DNA. It is noteworthy that an African *S*. *ruficorne* specimen (BB013) is embedded within *S*. *aureohirtum* clade. This topology likely results from a poor resolution of the nuclear phylogeny. Indeed, a visual inspection of the nuclear sequences further suggests that *S*. *ruficorne* BB013 belongs to the *S*. *ruficorne* clade with the presence of a single deletion detected only in *S*. *ruficorne* sequences (data not shown).

### Monophyly tests

The mitochondrial and nuclear phylogenies indicated that the three Reunion black fly species do not cluster together, suggesting that they originated through multiple colonizations (Figs 2 and 3). This hypothesis is corroborated by the results of monophyly tests based on mitochondrial and nuclear phylogenies, which rejected the monophyly of Reunion species (*H*_*1*_, posterior odd ≈ 0, Table 3).

**Table 3:** Results of monophyly tests based on posterior odds.

Monophyly hypothesis	Posterior probability P(H|D)		Posterior odd P(H|D)/ (1- P(H|D))	
	Mitochondrial	Nuclear	Mitochondrial	Nuclear
*H*_*1*_: Monophyly of Reunion species (*S*. *borbonense*, *S*. *ruficorne* and *S*. *triplex*)	< 1.1 x 10^−4^ (P)	< 2.2 x 10^−4^ (P)	< 1.1 x 10^−4^ (P)	< 2.2 x 10^−4^ (P)
	< 7.2 x10-5 (A)	< 2.0 x 10^−4^ (A)	< 7.2 x10^-5^ (A)	< 2.0 x 10^−4^ (A)
*H*_2_: Monophyly of *Simulium ruficorne*	< 1.1 x 10^−4^ (P)	0.096	< 1.1 x 10^−4^ (P)	0.106
	< 7.2 x10^-5^ (A)		< 7.2 x10^-5^ (A)	

(P), based on pseudo-ESS of tree topology; (A), based on approximate ESS of tree topology Warren et al. [59].

The mitochondrial phylogeny suggested that *S*. *ruficorne* is paraphyletic as some sequences obtained from Reunion *S*. *ruficorne* belong to the *S*. *borbonense* clade (Fig 2). This paraphyly was further supported by the results of monophyly tests, which rejected the monophyly of Reunion *S*. *ruficorne* (*H*_*2*_, posterior odd ≈ 0 for mitochondrial DNA and 0.11 for nuclear DNA, Table 3). All Reunion *S*. *ruficorne* were unambiguously identified as *S*. *ruficorne* based on morphology and harbored *S*. *ruficorne* nuclear material (ITS1 diagnosis). Altogether, these results indicate that Reunion black flies identified as *S*. *ruficorne* can also host *S*. *borbonense* haplotypes.

### Estimation of divergence times

The estimation of divergence times, based on the mutation rate of arthropods for mitochondrial DNA, showed that at least two Reunion black fly species diverged several million years before the emergence of Reunion Island (estimated at 2.1 Myr). Specifically, the divergence of *S*. *triplex* and the two other species from Reunion Island is estimated at 12.87 Myr ago (95% CI: 11.11–15.75), while *S*. *borbonense* and *S*. *ruficorne* diverged 6.46 Myr ago (95% CI: 4.71–8.30) (Fig 2). In contrast, *S*. *triplex* and specimens collected in Comoros diverged more recently: 0.67 Myr ago (95% CI: 0.31–1.11) (Fig 2). Interestingly, Reunion and African *S*. *ruficorne* diverged 2.24 Myr ago (95% CI: 1.46–3.04), which is close to the estimated age of Reunion Island emergence (Fig 2).

## Discussion

### Morphological and molecular identification of black fly species from Reunion and other Southwestern Indian Ocean islands

The investigation of Reunion black fly fauna allowed sampling three out of the four species described on the island: *Simulium ruficorne*, *Simulium borbonense* and *Simulium triplex*. The fourth species, *Simulium indoceanicum*, was not observed in our samples. The sampling was carried out during the austral summer and not through the whole year, thus we cannot rule out that the absence of *S*. *indoceanicum* resulted from either a restricted sampling period or geographic distribution of this species. Environmental perturbations are known to have considerable impact on black fly communities. These perturbations include hydrological modifications, deforestation, tourism development, habitat destruction and pollution [61,62]. Reunion Island has experienced a rapid economical development together with a fast increase of its demography (706 300 inhabitants in 1999 and 842 767 inhabitants in 2014 [63]) that involved important environmental perturbations. Islands are known to display high rates of species extinction [64] and data accumulated in the SWIO islands actually confirm a high proportion of extinctions, sometimes associated with human activities [65]. Thus, the extinction of *S*. *indoceanicum*, originally described from samples collected in 1983, can be hypothesized even though it remains difficult to address.

In addition to the investigation of Reunion black flies, our study brings some original information on regional black fly diversity. We describe *S*. *triplex* in the black fly fauna of Comoros archipelago. Consequently, *S*. *triplex* may not be considered as endemic to Mascarene archipelago anymore. On Seychelles archipelago, only one endemic *Simulium* species has been reported so far: *Simulium speculiventre* (Enderlein, 1914) belonging to the *ruficorne* species-group (*Nevermannia*) [39]. Thus, the taxonomy is consistent with our phylogenies (Figs 2 and 3) and specimens sampled from Seychelles archipelagos could effectively correspond to *S*. *speculiventre*.

The identification of black fly species from Reunion Island was thus far mainly based on morphological characters of larvae, pupae and the genitalia of male adult [47]. Although these characters are helpful, they require expertise and can be time-consuming notably for the genitalia dissection. Thus, we developed a molecular identification based on ITS1 length polymorphism that allows quick determination of Reunion black fly species and may be used to solve identification problems occurring with damaged specimens (S1 Fig). Such molecular diagnosis represents an important tool for taxonomic groups sheltering species that may be hard to distinguish on a strictly morphological basis [30,51]. These results also support the use of ITS1 as a molecular marker for closely related black fly species identification [50,51,54].

### Phylogeny of Reunion black fly species reveals *S*. *ruficorne* paraphyly and suggests mitochondrial introgression

Phylogenetic reconstructions allowed the description of Reunion black fly species relationships. Among the Reunion sampled species, *S*. *ruficorne* and *S*. *borbonense* are the most closely related species. Based on mitochondrial data, *S*. *ruficorne* appeared paraphyletic. Such paraphyly has been reported for other black fly species, complex species or species-group: *Simulium fenestratum* (Edwards, 1934) [66], *Simulium articum* (Malloch, 1914) [67] and *Simulium tuberosum* (Lundström, 1911) [68]. Interestingly, for these latter species, the paraphyly was supported by both mitochondrial and nuclear phylogenies. Here, the paraphyly of Reunion *S*. *ruficorne* is only supported by the mitochondrial phylogeny (Fig 2). Indeed, both the nuclear phylogeny and the ITS1 diagnostic concur to indicate that the Reunion *S*. *ruficorne* specimens harbouring *S*. *borbonense* mitotypes do possess *S*. *ruficorne* nuclear DNA. Such incongruence between mitochondrial and nuclear phylogenies has been previously reported within the *Simulium damnosum* complex (Theobald, 1903) [69] or other insect species [70]. Krueger and Hennings [69] proposed that such incongruence could result from mitochondrial introgression between black fly species evolving in sympatry. Interestingly, Conflitti et al. [67] also reported the paraphyly of sibling species within the *Simulium articum* complex. To explain this situation, the authors proposed possible introgressive hybridization with the sharing of alleles between members of sympatric species. In our case, the hypothesis of mitochondrial introgression requires interspecific hybridization between *S*. *borbonense* and Reunion *S*. *ruficorne*, two sympatric species belonging to the same species-group (*ruficorne*). Interestingly, this interspecific hybridization appears to be asymmetrical, and would result from interspecific crosses between Reunion *S*. *ruficorne* males and *S*. *borbonense* females. Indeed, no introgressed specimens descending from the reciprocal hybridization (*i*.*e*. Reunion *S*. *ruficorne* females with *S*. *borbonense* males) were observed. Such asymmetrical introgression has been previously reported for other organisms: birds, Flycather [71], spiders, Agelenidae [72], Honey Bee [73] and mosquitoes, *Culex pipiens* [74] and has been proposed to result from distinct processes such as differences in mate choice, non-reciprocal mechanical barriers, copulation, numerical dominance of one species over the other or *Wolbachia*-mediated selective sweep. To test this last hypothesis, the presence of *Wolbachia wsp* gene was investigated by using previously published PCR amplification test [75] and we could not detect *Wolbachia* in any of the tested flies (data not shown). The presence of paraphyletic species feeds the controversy on the use of mitochondrial markers alone to describe the taxonomy and phylogeny of invertebrates [76,77].

In this study, we estimated a divergence time of 6.46 Myr between *S*. *ruficorne* and *S*. *borbonense*, while the times of coalescence of all mitochondrial haplotypes for each of these two species are much more recent (< 1.00 Myr). Incomplete lineage sorting would be associated with more ancient mitochondrial lineages for each species although we cannot rule out possible colonization-dependent bottlenecks that would lead to the loss of a significant part of ancestral polymorphism. Interestingly, one nuclear sequence (18S/28S) was identically recovered from both African and Reunion *S*. *ruficorne* populations. This pattern is suggestive of a divergence time that is not long enough for lineage sorting to complete or gene flow between Reunion and African *S*. *ruficorne* populations.

### Multiple colonization events are responsible of Reunion black fly diversity

Speciation by geographical isolation following colonization is commonly accepted as a general source of endemism in oceanic island ecosystems [5,78]. The presence of black fly endemic species reported on remote oceanic islands suggests that speciation follows colonization events [41]. As far as Reunion species are concerned, a more complex scenario has been proposed according which the actual diversity results from successive colonizations from a single continental species (*i*.*e*. African *S*. *ruficorne*) [47]. Our phylogenetic reconstructions and monophyly tests support the hypothesis of multiple colonizations. *Simulium triplex* is present on different islands across the SWIO (Comoros archipelago, Mauritius Island and Reunion Island), suggesting that this species has a large spatial distribution and has potentially colonized Reunion Island from another island of the region. Altogether, we propose that the black fly diversity of Reunion Island has been built by a recurring influx of new colonists from the regional species pool, all belonging to *ruficorne* species-group.

In Pacific Ocean archipelagos, it has been shown that dispersal and speciation processes may explain the current diversity and distribution of black fly species. Within these islands, adaptive radiation represents the major mode of speciation with specialization of species to specific habitats [42,43,46,79]. Indeed, ecological adaptations has been proposed as an important factor of black fly diversification [66,68,79]. For example, on Society Islands, Joy, Craig and Conn [79] have proposed larval habitat shifts (cascades to rivers) as drivers of diversification between *Simulium oviceps* (Edwards, 1933) and *Simulium dussertorum* (Craig, 1997), two species belonging to the *oviceps* species-group (subgenus *Inseliellum*). Interestingly, Giudicelli [47] has shown that Reunion species exhibit differences in their distribution within rivers. Indeed, *S*. *triplex* is a stenothermic species, restricted to headwaters whereas *S*. *borbonense* and *S*. *ruficorne* are eurythermic species evolving within the lowland stream [47]. The implications of these ecological differences in the diversification of these black flies remain to be addressed but in any case our molecular dating does not support speciation within Reunion Island. However, we cannot rule out that *S*. *borbonense* and *S*. *triplex* result from speciation from a hypothetical *S*. *ruficorne* ancestor. Such speciation process could have taken place on nearby areas such as Mauritius Island, Rodrigues, Madagascar or Africa. In Madagascar, several black fly species are present and notably species belonging to the *ruficorne* species-group [39]. Such routes of colonization are possible but additional regional sampling is required to investigate these geographical origins. Altogether the Reunion black fly model highlights the importance of dispersal as a driver of arthropod diversity on a young Darwinian Island. The dispersal can be realized by the active flight of black flies or other mechanisms such as human activities, winds and hosts [35,41,42,80]. As far as Reunion species are concerned, dispersal with bird remains possible as the species of *ruficorne* species-group are known to be ornithophilic [47,81].

## Conclusions

In this study we showed that the Reunion black fly diversity resulted from multiple colonizations of distinct fly species rather than *in-situ* diversification, which improves our understanding of processes driving the biodiversity composition on young oceanic islands. Interestingly, we highlighted that two Reunion species, separated by approximately six million years of independent evolution, displayed an intriguing case of asymmetrical mitochondrial introgression. It is interesting to note that the evolutionary histories of some of these black flies’ hosts (*i*.*e*. *Zosterops* birds) and the blood parasites (*i*.*e*. *Leucocytozoon*) transmitted by black flies to these birds, have been investigated in the SWIO region [26–28]. The combination of these works and the present study can provide an interesting set of data to explore in detail the evolutionary histories of all partners evolving within a tripartite interaction on a young Darwinian Island.

## Supporting information

S1 Fig Migration results of ITS1—PCR products obtained from the different black fly species investigated in the present study. The colors red, blue, green and orange represent species from Morocco, Reunion Island, Comoros and Seychelles archipelagos, respectively. The electrophoresis was performed during 2 hours on a 2% agarose gel stained with 1X GelRed^TM^ (Biotium Inc.) The visualization was realized under UV.
(TIF)
