# Supplementary figures and images for: Evidence of multiple colonizations as a driver of black fly diversification in an oceanic island
Source: PLoS One. 2018 Aug 10;13(8):e0202015. doi: 10.1371/journal.pone.0202015 (PMC6086440; doi:10.1371/journal.pone.0202015)

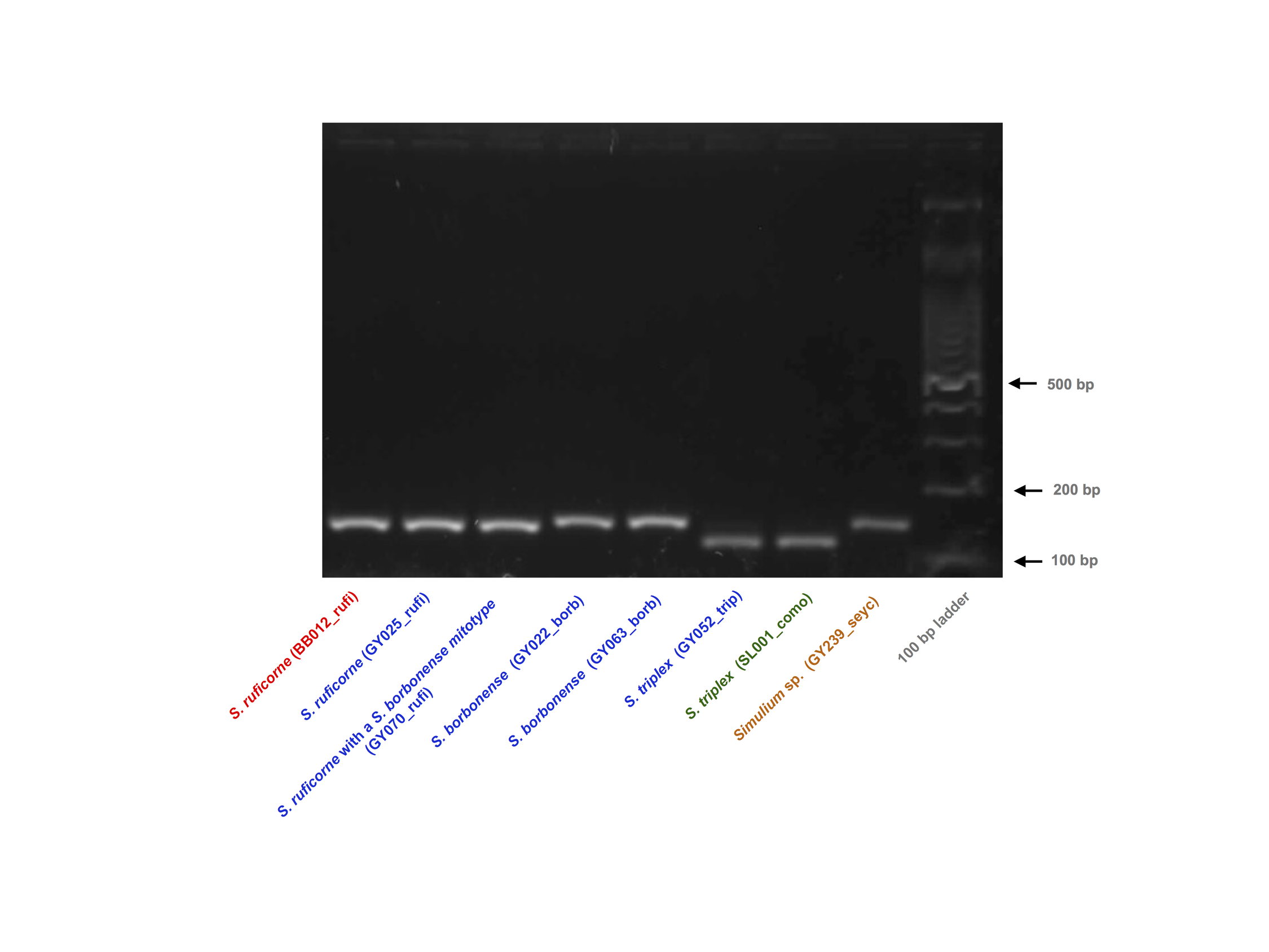

Supplement: S1 Fig — The colors red, blue, green and orange represent species from Morocco, Reunion Island, Comoros and Seychelles archipelagos, respectively. The electrophoresis was performed during 2 hours on a 2% agarose gel stained with 1X GelRedTM (Biotium Inc.) The visualization was realized under UV. (TIF) [file pone.0202015.s001.tif]
